# Spatial distribution of freshwater crustaceans in Antarctic and Subantarctic lakes

**DOI:** 10.1038/s41598-019-44290-4

**Published:** 2019-05-28

**Authors:** Angie Díaz, Claudia S. Maturana, Luz Boyero, Patricio De Los Ríos Escalante, Alan M. Tonin, Francisco Correa-Araneda

**Affiliations:** 1 Departamento de Zoología, Facultad de Ciencias Naturales y Oceanográficas, Universidad de Concepción, Concepción, Chile; 2 Instituto de Ecología y Biodiversidad (IEB), Las Palmeras 3425, Ñuñoa, Santiago Chile; 3 Laboratorio de Ecología Molecular, Departamento de Ciencias Ecológicas, Facultad de Ciencias, Universidad de Chile, Las Palmeras 3425, Ñuñoa, Santiago Chile; 4 Department of Plant Biology and Ecology, Faculty of Science and Technology, University of the Basque Country (UPV/EHU), Leioa, Spain; 5 IKERBASQUE, Bilbao, Spain; 6 Departamento de Ciencias Biológicas y Químicas, Facultad de Recursos Naturales, Universidad Católica de Temuco, Temuco, Chile; 7 Núcleo de Investigación en Estudios Ambientales y Departamento de Ciencias Ambientales (Facultad de Recursos Naturales), Universidad Católica de Temuco, Temuco, Chile; 8 Department of Ecology, IB, Universidade de Brasília, Brasília Distrito Federal, Brazil; 9 Unidad de Cambio Climático y Medio Ambiente, Instituto de Estudios del Hábitat, Facultad de Arquitectura y Construcción, Universidad Autónoma de Chile, Temuco, Chile

**Keywords:** Biogeography, Limnology

## Abstract

Antarctic and Subantarctic lakes are unique ecosystems with relatively simple food webs, which are likely to be strongly affected by climate warming. While Antarctic freshwater invertebrates are adapted to extreme environmental conditions, little is known about the factors determining their current distribution and to what extent this is explained by biogeography or climate. We explored the distribution of freshwater crustaceans (one of the most abundant and diverse group of organisms in Antarctic and Subantarctic lakes) across four biogeographic provinces (Continental Antarctic, CA; Maritime Antarctic, MA; Subantarctic islands, SA; and Southern Cool Temperate, SCT) based on the literature, predicting that species distribution would be determined by biogeography, spatial autocorrelation among regions (in relation to dispersal) and climate. We found that variation in species composition was largely explained by the joint effect of spatial autocorrelation and climate, with little effect of biogeography – only regions within the SA province had a clearly distinct species composition. This highlights a plausible main influence of crustacean dispersal – mainly through migratory seabirds – and suggests that some regions will be more affected by climate warming than others, possibly in relation to the existence of nearby sources of colonists.

## Introduction

Antarctica is the Earth’s southernmost continent, almost entirely covered by an ice sheet. Remarkably, however, it holds a high variety of lake ecosystems, many located in ice-free coastal areas, and some in ice-free inland areas and in surrounding Antarctic and Subantarctic islands^1^. These lakes are characterized by their low metazoan diversity and low food-web complexity, with higher trophic levels such as fish being missing or largely absent^1–3^. Such low diversity and ecological complexity could make these ecosystems particularly vulnerable to ecological changes as a result of climate change-driven extinctions^4^. It is thus important to explore distribution patterns of their biota and the determinants of such patterns, which can shed light on future ecological changes^5^.

Crustaceans are the most diverse and well-documented freshwater invertebrates in Antarctic and Subantarctic lakes, where the eight major crustacean orders are represented^6^. Most taxa are common components of zooplankton, where they occupy a wide range of ecological niches, and can respond quickly to environmental change, including temperature increase^7^. Thus, they are considered sentinel organisms which can help understanding climate change effects^8,9^. The occurrence of crustacean species in Antarctic and Subantarctic lakes has been reported in many publications and compiled in two major reviews^6,10^, but no attempt has been made to explore whether their distribution is explained mostly by biogeography or whether climate is a main determinant. We explored this question using published information and tested the hypothesis that variation in crustacean species composition across regions within Antarctica is determined by (i) biogeography (i.e., regions from the same biogeographic province will have more similar species composition than regions from different provinces), (ii) spatial autocorrelation among regions (i.e., regions closer to each other will have more similar species composition than more distant regions due to higher dispersal among them) and (iii) climate (due to species-specific environmental constraints).

## Results

We extracted a list of 66 crustacean taxa (59 species and 7 genera/mosphospecies; hereafter species for simplicity) representing 8 orders (Table 1). Species were distributed mainly across the Subantarctic islands (SA) and Southern Cool Temperate province (SCT) (46 and 26 species, respectively). SA showed at least 1 species from each of the 8 crustacean orders, and all SA islands except Prince Edward Island (Pe) contained species that were endemic of our study area. Iles Kerguelen (Kr) showed the greatest richness (19 species, 5 endemic), followed by South Georgia (Sg; 17 species, 5 endemic), Macquarie Island (Mc; 14 species, 7 endemic including *Iais sp*., the only isopod recorded at these latitudes) and Iles Crozet (Cr; 11 species, 2 endemic). For SCT, Falkland/Malvinas Islands (Fa) concentrated most of the species (25) allocated across 7 orders and a high number of endemic species (10). Campbell (Ca) and Auckland Islands (Ak) had one endemic species, *Chiltonia mihiwaka* (Amphipoda). The cladoceran *Ovalona weinicki*^11^ and the calanoid *Boeckella poppei*^12^ were present in most localities (8 and 6, respectively), but only *B*. *poppei* was present in all of them. In the Maritime Antarctic province (MA) there were 9 species across 4 orders. South Orkney Islands (So) was the richest region, with all 9 species and the only records of Podocopida and Cladocera within MA, with the exception of *Macrothrix oviformis* and *O*. *weinicki*, which were also found in South Shetland Islands (Ss) and Antarctic Peninsula (Pa). In the Continental Antarctic province (CA) there were 7 species of the orders Cladocera [*Daphniopsis studeri* at Enderby (En)], Calanoida [*B*. *poppei* at En and *Gladioferens antarcticus* at Wilkes (Wi)] and Cyclopoida (*Diacyclop* sp. in all 3 regions), with 3 species endemic of this province (*D*. *joycei*, *D*. *kaupi* and *D*. *walkeri*).

**Table 1:** Presence/absence matrix of crustacean taxa in lakes of each study region based on Pugh *et al*. 2002 (1) and Dartnall *et al*. 2017 (2).

Order/*Species*		CA		MA			SA						SCT			
	En	Wi	Sc	So	Ss	Pa	Sg	Pe	Cr	Kr	Hd	Mc	Fa	Ca	Ak	Ref.
**Anostraca**																
*Branchinecta gaini* (Daday, 1910)*				•	•	•	•						•			^1,2^
**Cladocera**																
*Alona guttata* (Sars, 1862)**													•			^1^
*Alona quadrangularis* (O.F. Müller, 1776)**												•				^1,2^
*Camptocercus aloniceps* (Ekman, 1900)*							•						•			^1,2^
*Camptocercus rectirostris* (Schödler, 1862)							•									^1^
*Chydorus patagonicus* (Ekman, 1900)							•					•				^1,2^
*Chydorus sphaericus* (O.F. Müller, 1776)*				•			•		•	•			•			^1,2^
*Daphnia gelida* (Brady, 1918)**												•				^2^
*Daphnia pulex* (Leydig, 1860)													•			^1^
*Daphniopsis studeri* (Rühe, 1914)*	•							•	•	•	•					^1,2^
*Ceriodaphnia silvestrii* (Daday, 1902)**													•			^1^
*Ilyocryptus brevidentatus* (Ekman, 1905)				•			•						•			^1,2^
*Macrothrix boergeni* (Studer, 1878)**										•						^2^
*Macrothrix flagellata* (Smirnov & Timms, 1983)**												•				^2^
*Macrothrix laticornis* (Jurine, 1820)**													•			^1^
*Macrothrix ruehei* (Kotov, 2007)								•	•							^2^
*Macrothrix oviformis* (Ekman, 1900)*				•	•	•	•									^2^
*Macrothrix* sp. (Baird, 1843)*											•					^2^
*Ovalona weinecki* (Studer, 1878)*				•	•		•	•		•	•	•	•			^2^
*Pleuroxus macquariensis* (Frey, 1993)**												•				^1,2^
*Pleuroxus wittsteini* (Studer, 1878)								•		•	•					^1,2^
*Bosmina coregoni* (Baird, 1857)													•			^2^
**Podocopida**																
*Candona* sp. (Baird, 1845)							•									^1,2^
*Chlamydotheca pestai* (Graf, 1931)**							•									^1,2^
*Chlamydotheca symmetrica* (Vávra, 1898)**													•			^1^
*Cypretta* sp. (Vávra, 1895)**												•				^1,2^
*Eucypris corpulenta* (G. O. Sars, 1895)**										•						^1,2^
*Eucypris fontana* (Graf, 1931)*				•			•									^1,2^
*Eucypris virens* (Jurine, 1820)										•		•				^1,2^
*Ilyodromus kerguelensis* (G.W. Müller, 1906)								•	•	•						^1,2^
*Neocypridopsis frigogena* (Graf, 1931)*				•			•									^1,2^
*Tanycypris* sp. (Triebel, 1959)							•									^1,2^
*Candonopsis falklandica* (Vávra, 1898)**													•			^1^
*Newnhamia patagonica* (Vávra, 1898)**													•			^1^
**Calanoida**																
*Boeckella poppei* (Mrázek, 1901)*	•			•	•	•	•						•			^1,2^
*Boeckella michaelseni* (Mrázek, 1901)*							•						•			^1,2^
*Boeckella brevicaudata* (Brady, 1875)										•	•	•				^1,2^
*Boeckella vallentini* (Scott T., 1914)*								•	•	•			•			^1,2^
*Boeckella* sp. (Guerne & Richard, 1889)*			•													^2^
*Gladioferens antarcticus* (Bayly, 1994)**		•														^1,2^
*Parabroteas sarsi* (Daday, 1901)*				•		•	•						•			^1,2^
**Cyclopoida**																
*Acanthocyclops robustus* (Sars G.O., 1863)**										•						^1,2^
*Acanthocyclops vernalis* (Fischer, 1853)										•						^1,2^
*Diacyclops michaelseni* (Mrázek, 1901)**										•			•			^1,2^
*Diacyclops mirnyi* (Borutzky & Vinogradov, 1957)**	•	•											•			^1,2^
*Diacyclops joycei* (Karanovic *et al*. 2014)**			•													^2^
*Diacyclops kaupi* (Karanovic *et al*. 2014)**		•														^2^
*Diacyclops walkeri* (Karanovic *et al*. 2014)**	•															^2^
*Mixocyclops crozetensis* (Kiefer, 1944)**									•							^1,2^
*Paracyclops chiltoni* (Thomson G.M., 1883)									•	•						^1,2^
*Tropocyclops prasinus prasinus* (Fischer, 1860)													•			^1^
**Harpacticoida**																
*Antarctobiotus koenigi* (Pesta, 1928)**							•									10,6
*Antarctobiotus robustus* (Richters, 1907)									•	•						10,6
*Epactophanes richardi* (Mrázek, 1893)								•	•	•	•					10,6
*Marionobiotus jeanneli* (Chappuis, 1940)*								•		•		•				10,6
*Marionobiotus sp*. (Chappuis, 1940)*												•				6
*Tigriopus angulatus* (Lang, 1933)*								•	•	•		•				10,6
*Attheyella (D*.*) trigonura* (Eckman, 1905)													•			10
**Amphipoda**																
*Kergueleniola macra* (Ruffo, 1970)**										•						10,6
*Pseudingolfiella possessionis* (Smet, 2015)**									•							6
*Chiltonia mihiwaka* (Chilton, 1898)														•	•	10
*Hyalella curvispina* (Shoemaker, 1942)													•			10
*Hyalella neonoma* (Stock & Platvoet, 1991)													•			10
*Falklandella obtusa* (Schellenberg, 1931)													•			10
*Praefalklandella cuspidatus* (Schellenberg, 1931)**													•			10
**Isopoda**																
*Iais* sp. (Bovallius, 1886)												•				6
N° total species	4	3	1	9	4	4	17	9	11	19	6	14	25	1	1	
N° species by zones	7	9	46	26												
N° order	3	4	8	7												

Provinces: CA, Continental Antarctic; MA, Maritime Antarctic; SA, Subantarctic islands; SCT, Southern Cool Temperate. Regions: En, Enderby; Wi, Wilkes; Sc, Scott; So, South Orkney Islands; Ss South Shetland Islands; Pa, Antarctic Peninsula; Sg, South Georgia; Pe, Prince Edward Island; Cr, Iles Crozet; Kr, Iles Kerguelen; Hd, Heard Island; Mc, Macquarie Island; Fa, Falkland/Malvinas Islands; Ca, Campbell Island; Ak, Auckland Island. Underlined species: Endemic from one biogeographic province; *: Endemic from two or more biogeographic provinces; **: Endemic from one region within a province.

**Figure 1:**
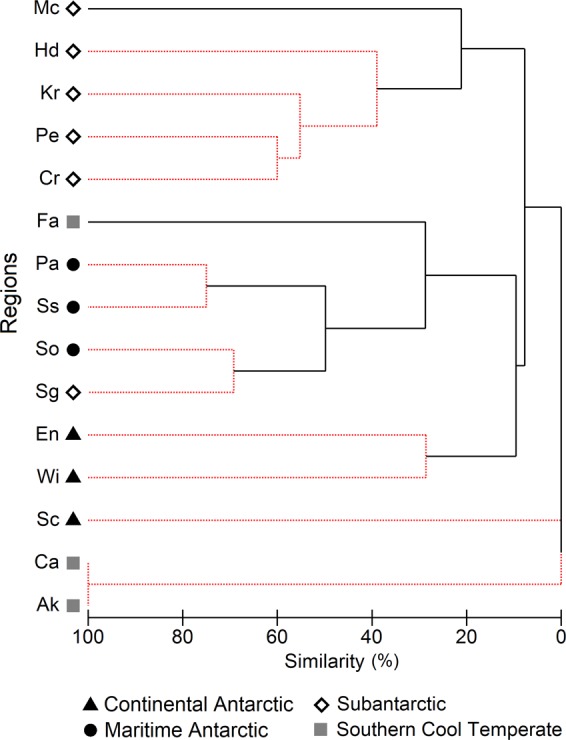
Results of hierarchical cluster analysis grouping the study regions based on crustacean species composition. Regions: see Table 1 footnote.

**Figure 2:**
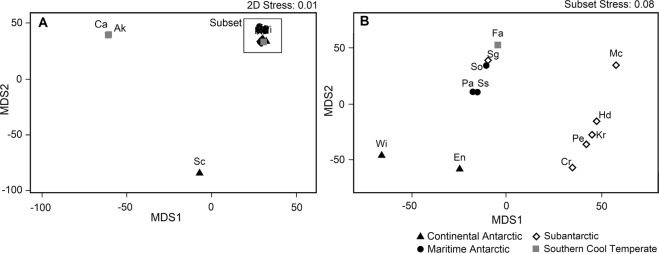
Results of NMDS ordination of study regions based on crustacean species composition (**A**), with inset of the main group (**B**). Regions: see Table 1 footnote.

Cluster analysis showed 3 distinct groups of regions according to crustacean species composition: (1) Ca and Ak from SCT; (2) Sc from CA; and (3) all regions from SA, MA and CA (excluding En and Wi) and Fa from SCT. The latter group was further divided into 3 sub-groups: (3a) Cr, Pe, Kr, Heard Island (Hd) and Mc from SA, the latter without significant support; (3b) MA, Sg from SA and Fa from SCT, the latter without significant support; and (3c) En and Wi from CA (Fig. 1). The NMDS produced the same groups as cluster analysis (Fig. 2).

ANOSIM showed significant differences among biogeographic provinces (Global *R* = 0.57, *p* = 0.001). Pairwise tests showed significant differences for CA vs. SA, MA vs. SA and SA vs. SCT and no significant differences among CA, MA and SCT (Table 2), thus revealing two groups (Group 1: SA; Group 2: CA, MA and SCT). Based on similarity percent analysis (SIMPER), the species that most contributed to Group 1 were *Ovalona* *weinecki* (19.5%), *Epactophanes richardi* (13.6%), *Daphniopsis* *studeri* (13.6%) and *Tigriopus angulatus* (11.1%); species that most contributed to Group 2 were *Boeckella* *poppei* (30.2%), *Chiltonia* *mihiwaka* (20.4%) and *Branchinecta gaini* (15.4%); dissimilarity between Group 1 and Group 2 was explained by a large number of species, all with lower contribution values (<5.2%) (Table 3).

**Table 2:** ANOSIM Pairwise test analysis between provinces based on the presence/absence crustacean matrix. Provinces: see Table 1 footnote.

	Permutations	*R*	*p-value*
CA vs. MA	10	0, 556	0, 10
CA vs. SA	84	0, 698	0, 01
CA vs. SCT	10	0, 185	0, 20
MA vs. SA	84	0, 497	0, 04
MA vs. SCT	10	0, 556	0, 10
SA vs. SCT	84	0, 694	0, 02

**Table 3:** Similarity Percent analysis (SIMPER) to identify the contribution (%) of each species to the similarity and dissimilarity of each group.

	Average Abundance		Similarity Contribution		Dissimilarity Contribution
	Group 1	Group 2	Group 1	Group 2	Group 1 vs Group 2
*Epactophanes richardi*	0, 67	—	13, 57	0	5, 26
*Daphniopsis studeri*	0, 67	0, 11	13, 57	0	4, 89
*Ovalona weinecki*	0, 83	0, 33	19, 47	5, 75	4, 79
*Tigriopus angulatus*	0, 67	0	11, 11	0	4, 37
*Pleuroxus wittsteini*	0, 5	0	6, 79	0	4, 06
*Boeckella brevicaudata*	0, 5	0	5, 74	0	3, 65
*Boeckella vallentini*	0, 5	0, 11	5, 68	0	3, 38
*Ilyodromus kerguelensis*	0, 5	0	5, 68	0	3, 38
*Boeckella poppei*	0, 17	0, 56	0	30, 17	3, 28
*Marionobiotus jeanneli*	0, 5	0	5, 22	0	3, 17
*Chydorus sphaericus*	0, 5	0, 22	4, 62	0	2, 93
*Branchinecta gaini*	0, 17	0, 44	0	15, 41	2, 69
*Macrothrix ruehei*	0, 33	0	0	0	2, 6
*Macrothrix oviformis*	0, 17	0, 33	0	11, 39	2, 46
*Parabroteas sarsi*	0, 17	0, 33	0	0	2, 09
*Chiltonia mihiwaka*	0	0, 22	0	20, 42	2
*Antarctobiotus robustus*	0, 33	0	0	0	1, 97
*Paracyclops chiltoni*	0, 33	0	0	0	1, 97
*Diacyclops mirnyi*	0	0, 33	0	8, 7	1, 95
Cumulative Contribution			91, 45	91, 84	60, 89

**Figure 3:**
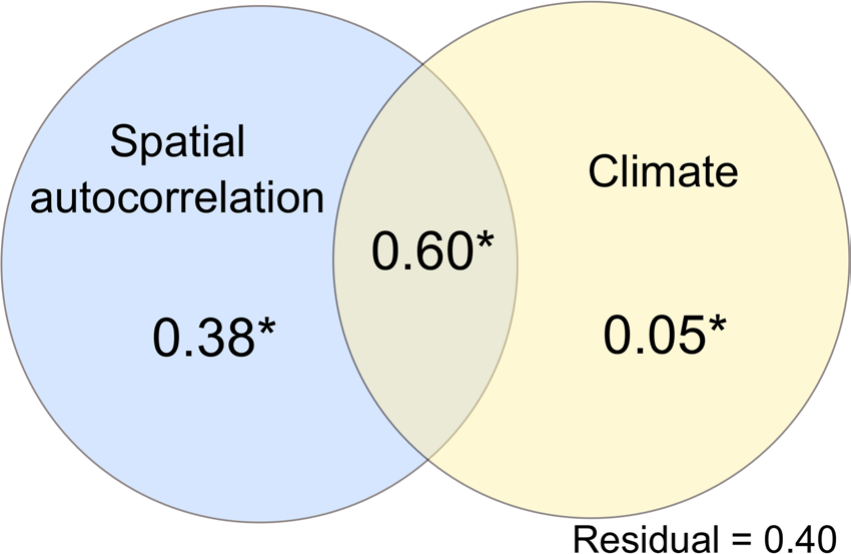
Results of partial redundancy analysis (pRDA) showing the amount of variability in crustacean distribution attributable to spatial autocorrelation among regions, climate, and the shared contribution of both variables. The amount of variability explained by each factor or their shared contribution is based on R^2^adj; asterisks indicate significant results (at p < 0.05, based on 999 permutations).

The partial redundancy analysis (pRDA) showed that both spatial autocorrelation and climate explained a significant part of the variance (spatial autocorrelation: R^2^_adj_ = 0.38, p = 0.003, variance explained = 26.67%; climate: R^2^_adj_ = 0.05, p = 0.029, variance explained = 3.73%), but most variance was due to the shared contribution of both variables (R^2^_adj_ = 0.60, p = 0.001, variance explained = 41.87). Residuals explained 27% of the variance (R^2^_adj_ = 0.40) (Fig. 3).

## Discussion

Our results showed that spatial autocorrelation among Antarctic and Subantarctic lakes and climate were key determinants of crustacean distribution, while biogeography had a secondary role. Multivariate analyses revealed that only the Subantarctic biogeographic province had a distinct crustacean fauna. This province contained 46 species belonging to the 8 crustacean orders described for Antarctica. The species that most contributed to the distinctness of the Subantarctic province was the cladoceran *O*. *weinecki*^11^, which is the only Antarctic representative of a genus of mainly tropical and subtropical distribution^13^. Van Damme and Dumont^14^ re-described this species from a complex of *Alona* sp. (principally, *A*. *weinecki*) described for Subantarctic islands^6,10^.

Geographic distance may explain the fact that Macquarie island, which is separated ~6,000 km from the other Subantarctic islands, shared only a few species with them; and could also explain the high incidence of endemic species of crustaceans and other freshwater organisms in Macquarie island^15^. The relevance of distance was also revealed by our partial redundancy analysis (which showed that spatial autocorrelation among regions explained a large amount of variance), and it is most likely related to patterns of dispersal. Dispersal among nearby islands occurs mainly via migratory seabirds, which can transport resistant eggs within the gut or in mud adhering to feet^1,6,10,16,17^. Distance among regions is often a key determinant in the distribution of freshwater fauna^18^, which may help explain some inconsistencies in the definition of biogeographic provinces.

The Maritime Antarctic province had similar species composition to South Georgia island from the Subantarctic province and the South American Falkland/Malvinas islands from the Southern Cool Temperate province. All these regions are separated by less than 2,000 km, so geographic distance could again be important in their similarity. It has also been proposed that Antarctic and South American crustacean fauna could have a common origin, as both continents were separated ~30 Mya^19,20^, thus being vicariant faunas^21,22^. However, this is unlikely, because most crustacean species in Continental and Maritime Antarctic provinces are Holocene immigrants, having arrived within the last 11 ka^10^.

Campbell and Auckland Islands (New Zealand), from the Southern Cool Temperate province, had a distinct fauna and mainly shared the unique amphipod species *C*. *mihiwaka*^23^ and the widespread *B*. *poppei*. The separation of the Scott sector from the Continental Antarctic province from other regions was related to the Cyclopoida *D*. *joycei*^24^, which is the only species that inhabits this region. The other two sectors of the Continental Antarctic province (Enderby and Wilkes) have other species of this genus: *D*. *mirnyi* (present in both regions), *D*. *walker* (in Enderby) and *D*. *kaupi* (in Wilkes). This group of *Diacyclops* species is known as the “michaelseni group”^25^, a circum-Antarctic assemblage that shares some morphological characteristics and originated in Antarctic freshwater lakes in late Pliocene, prior to the onset of glaciation^24^. Lastly, the wide distribution of some species such as *O*. *weinecki* or *B*. *poppei* could be due to recent colonization events from northern latitudes, of anthropogenic origin in some cases^10^. Other authors have suggested an ancient origin for these species, which may have survived during Pleistocene glaciations in refugia^26–28^, such as Kerguelen Island^14^.

The lack of consistent climatic data for different Antarctic and Subantarctic regions precluded a more robust assessment of the influence of climate on freshwater crustacean distribution. However, our analyses using latitude as surrogate for climate suggested that climate affects distributional patterns, and its effect is variable among regions, depending on their location. Thus, some regions of Antarctica are likely to be more affected by climate warming than others, and this variation could be related to geographic distance to other sources of colonists. These differences may be further enhanced by the fact that some parts of Antarctica are experiencing greater temperature increases than others; the increase is particularly large in the Antarctic Peninsula, which has registered an increase of 0.67 °C per decade in the last 50 years^29–31^. Further studies are needed in order to improve our knowledge on biodiversity patterns and their main drivers in this continent that is experiencing some of the most rapid environmental changes on Earth^32^.

## Methods

### Study area

**Figure 4:**
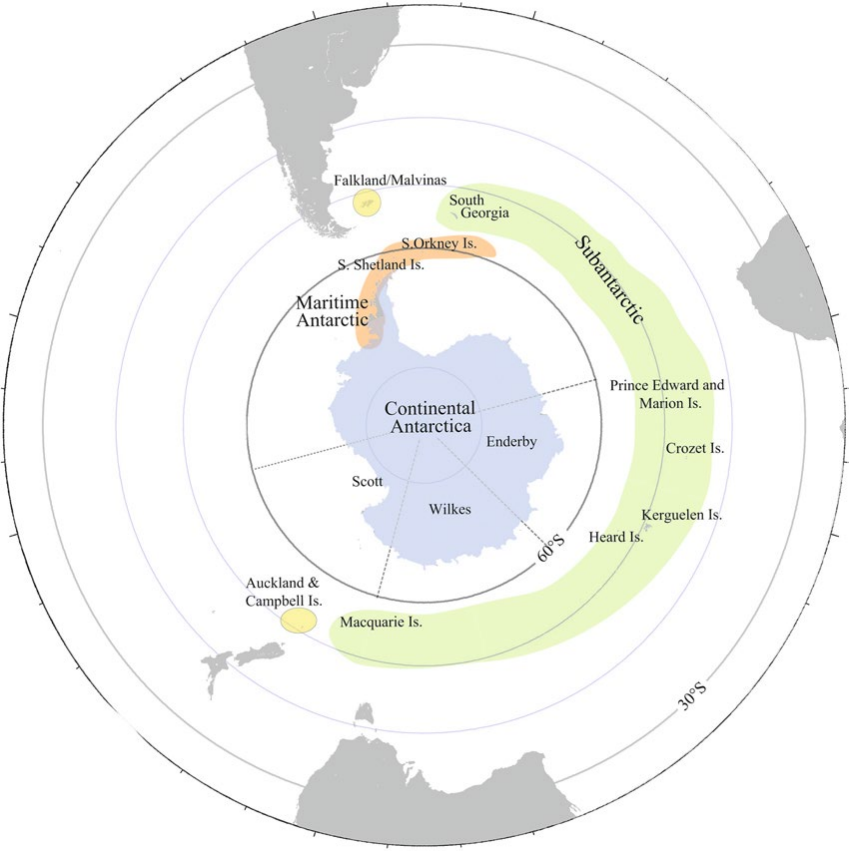
Map of the four Antarctic and Subantarctic biogeographic provinces considered in this study: Continental Antarctic (in blue colour), Maritime Antarctic (orange), Subantarctic islands (green) and Southern Cool Temperate (yellow).

The Antarctic continent can be divided into 3 biogeographic provinces which differ considerably in climatic conditions^3,32–34^: the CA, which is the largest and coldest region with temperature rarely above freezing^35^, comprising the continent landmass south of 72°S and the Balleny Islands; the MA, which includes the western side of the Antarctic Peninsula north of 72°S and experiences seasonal snowmelt^35^; and the SA, which comprises a series of islands and small archipelagos in the Southern Ocean proximate to the zone of Antarctic Polar Front (APF), with temperatures that on average are above freezing point year-round^36^. Besides, we considered a fourth biogeographic province, north of the APF and influenced by low temperatures: the SCT province, which is formed by several islands from New Zealand and South America^10^, with cool to cold temperate climate^37^ (Fig. 4).

### Data collection

We elaborated a presence/absence matrix of all freshwater crustacean species reported for Antarctic and Subantarctic lakes, based on two major literature reviews^6,10^, which contained all the available information to date. We divided each biogeographic province into regions following the above two reviews: CA comprised the En (30°E–90°E), Wi (90°E–150°E) and Sc (150°E–150°W) sectors; MA included the Pa, Ss and So; SA included Sg, Pe, Mc, Hd, Cr and Kr; and SCT included Ca and Ak from New Zealand and Fa from South Atlantic ocean (Table 1, Fig. 4). We excluded suspect records from the dataset, ruled out possible synonymies, and updated scientific names. We assumed that sampling effort of different taxa was similar across sites, although potential differences may have some influence on our results.

### Data analysis

We explored the influence of biogeography on regional species composition using hierarchical cluster analysis integrated with similarity profile analysis in SIMPROF^38^ and metric multidimensional scaling, MDS^39^ based on a similarity matrix using the Jaccard index. We tested for significance of the different groups of regions generated by cluster analysis using one-way ANOSIM, with biogeographic province as factor^40,41^, followed by pairwise tests. Further, we identified the main species associated with each group through SIMPER based on the presence/absence matrix of crustacean species. These analyses were done using Primer v.6 software^42^.

We explored the separate and joint influence of spatial autocorrelation among regions and climate using pRDA. The amount of variation explained by each factor and by their shared contribution was calculated by variance partitioning analysis, which is based on adjusted R^2^ (R^2^_adj_), and their statistical significance tested through permutation tests (999 randomizations). Species composition data was Hellinger-transformed prior to analysis to provide an unbiased estimate of variance partitioning based on RDA. Spatial autocorrelation was obtained with the eigenfunction analysis known as Principal Coordinates of Neighbor Matrix PCNM^43^, which created 10 spatial variables (PCNM vectors) based on a matrix of Euclidean distances between regions calculated using the geographic coordinates. These vectors allow the representation of different spatial relationships among regions at different spatial scales and can be treated as independent variables^44^. As we were not able to obtain consistent climatic data for all the study regions – there are relatively few meteorological stations in Antarctica and any gross estimate based on different data sources could be misleading –, we used decimal latitude as surrogate for climate. To eliminate any effect caused by different elevations, we used the residuals of a linear regression with latitude (as a response variable) against elevation (as a predictor) in the analysis^45,46^. Elevation was obtained from www.gps-coordinates.net based on latitude and longitude. These analyses were performed on R v. 3.5.1^47^, using the functions rda, varpart, anova.cca and pcnm from vegan package^48^.

## Data Availability

Data will be available on the Open Science Framework online repository.
