# Publisher Correction: Spatial distribution of freshwater crustaceans in Antarctic and Subantarctic lakes

**DOI:** 10.1038/s41598-020-58516-3

**Published:** 2020-01-30

**Authors:** Angie Díaz, Claudia S. Maturana, Luz Boyero, Patricio De Los Ríos Escalante, Alan M. Tonin, Francisco Correa-Araneda

**Affiliations:** 1 Departamento de Zoología, Facultad de Ciencias Naturales y Oceanográficas, Universidad de Concepción, Concepción, Chile; 2 Instituto de Ecología y Biodiversidad (IEB), Las Palmeras, 3425 Ñuñoa, Santiago Chile; 3 Laboratorio de Ecología Molecular, Departamento de Ciencias Ecológicas, Facultad de Ciencias, Universidad de Chile, Las Palmeras, 3425 Ñuñoa, Santiago Chile; 4 Department of Plant Biology and Ecology, Faculty of Science and Technology, University of The Basque Country (UPV/eHU), Leioa, Spain; 5 IKERBASQUE, Bilbao, Spain; 6 Departamento de Ciencias Biológicas y Químicas, Facultad de Recursos Naturales, Universidad Católica de Temuco, Temuco, Chile; 7 Núcleo de Investigación en Estudios Ambientales y Departamento de Ciencias Ambientales (facultad de Recursos naturales), Universidad católica de Temuco, Temuco, Chile; 8 Department of Ecology, IB, Universidade de Brasília, Brasília, Distrito federal Brazil; 9 Unidad de Cambio Climático y Medio Ambiente, Instituto de Estudios del Hábitat, Facultad de Arquitectura y Construcción, Universidad Autónoma de Chile, Temuco, Chile

Correction to: *Scientific Reports* 10.1038/s41598-019-44290-4, published online 28 May 2019

This Article contains errors in Table 1. In column 17 ‘Ref.’, all instances of ‘1’ should read ‘10’. In addition, all instances of ‘2’ should read ‘6’.

As a result, in the legend for Table 1,

‘Presence/absence matrix of crustacean taxa in lakes of each study region based on Pugh *et al*. 2002 (1) and Dartnall *et al*. 2017 (2)’

should read:

‘Presence/absence matrix of crustacean taxa in lakes of each study region based on Pugh *et al*. 2002 (10) and Dartnall *et al*. 2017 (6).’

The correct Table 1 appears below.

**Table 1:** Presence/absence matrix of crustaceans in lakes of each study province (CA, Continental Antarctic; MA, Maritime Antarctic; SA, Subantarctic islands; SCT, Southern Cool Temperate), based on Pugh *et al*. 2002^10^ and Dartnall *et al*. 2017^6^.

Order/*Specie*	CA			MA			SA						SCT			Ref.
	En	Wi	Sc	So	Ss	PA	Sg	Pe	Cr	Kr	Hd	Mc	Fa	Ca	Ak	
**Anostraca**																
*Branchinecta gaini*				●	●	●	●						●			^10, 6^
**Cladocera**																
*Alona guttata*													●			^10^
*Alona quadrangularis*												●				^10, 6^
*Camptocercus aloniceps*							●						●			^10, 6^
*Camptocercus rectirostris*							●									^10^
*Chydorus patagonicus*							●					●				^10, 6^
*Chydorus sphaericus*				●			●		●	●			●			^10, 6^
*Daphnia gelida*												●				^6^
*Daphnia pulex*													●			^10^
*Daphniopsis studeri*	●							●	●	●	●					^10, 6^
*Ceriodaphnia silvestrii*													●			^10^
*Ilyocryptus brevidentatus*				●			●						●			^10, 6^
*Macrothrix boergeni*										●						^6^
*Macrothrix flagellata*												●				^6^
*Macrothrix laticornis*													●			^10^
*Macrothrix ruehei*								●	●							^6^
*Macrothrix oviformis*				●	●	●	●									^6^
*Macrothrix sp*.											●					^6^
*Ovalona weinecki*				●	●		●	●		●	●	●	●			^6^
*Pleuroxus macquariensis*												●				^10, 6^
*Pleuroxus wittsteini*								●		●	●					^10, 6^
*Bosmina (Eubosmina) coregoni*													●			^10^
**Podocopida**																
*Candona sp*.							●									^10, 6^
*Chlamydotheca pestai*							●									^10, 6^
*Chlamydotheca symmetrica*													●			^10^
*Cypretta cf seurati*												●				^10, 6^
*Eucypris corpulenta*										●						^10, 6^
*Eucypris fontana*				●			●									^10, 6^
*Eucypris virens*										●		●				^10, 6^
*Ilyodromus kerguelensis*								●	●	●						^10, 6^
*Neocypridopsis frigogena*				●			●									^10, 6^
*Tanycypris sp*.							●									^10, 6^
*Candonopsis falklandica*													●			^10^
*Newnhamia patagonica*													●			^10^
**Calanoida**																
*Boeckella poppei*	●			●	●	●	●						●			^10, 6^
*Boeckella michaelseni*							●						●			^10, 6^
*Boeckella brevicaudata*										●	●	●				^10, 6^
*Boeckella vallentini*								●	●	●			●			^10, 6^
*Boeckella sp*.												●				^6^
*Gladioferens antarcticus*		●														^10, 6^
*Parabroteas sarsi*				●		●	●						●			^10, 6^
**Cyclopoida**																
*Acanthocyclops michaelseni*										●			●			^10, 6^
*Acanthocyclops robustus*										●						^10, 6^
*Acanthocyclops vernalis*										●						^10, 6^
*Diacyclops mirnyi*	●	●											●			^10, 6^
*Diacyclops joycei*			●													^6^
*Diacyclops kaupi*		●														^6^
*Diacyclops walkeri*	●															^6^
*Mixocyclops crozetensis*									●							^10, 6^
*Paracyclops chiltoni*									●	●						^10, 6^
*Tropocyclops prasinus*													●			^10^
**Harpacticoida**																
*Antarctobiotus koenigi*							●									^10, 6^
*Antarctobiotus robustus*									●	●						^10, 6^
*Epactophanes richardi*								●	●	●	●					^10, 6^
*Marionobiotus jeanneli*								●		●		●				^10, 6^
*Marionobiotus sp*.												●				^6^
*Tigriopus angulatus*								●	●	●		●				^10, 6^
*Attheyella (D.) trigonura*													●			^10^
**Amphipoda**																
*Kergueleniola macra*										●						^10, 6^
*Pseudingolfiella possessionis*									●							^6^
*Chiltonia mihiwaka*														●	●	^10^
*Hyalella curvispina*													●			^10^
*Hyalella neonoma*													●			^10^
*Falklandella obtusa*													●			^10^
*Praefalklandella cuspidatus*													●			^10^
**Isopoda**																
*Lais sp*.												●				^6^
Nº total species	4	3	1	9	4	4	17	9	11	19	6	14	25	1	1	
Nº species by zones	7			9			46						26			
Nº order	3			4			8						7			

