# Author Correction: Publisher Correction: Spatial distribution of freshwater crustaceans in Antarctic and Subantarctic lakes

**DOI:** 10.1038/s41598-021-97702-9

**Published:** 2021-09-10

**Authors:** Angie Díaz, Claudia S. Maturana, Luz Boyero, Patricio De Los Ríos Escalante, Alan M. Tonin, Francisco Correa-Araneda

**Affiliations:** 1 Departamento de Zoología, Facultad de Ciencias Naturales y Oceanográficas, Universidad de Concepción, Concepción, Chile; 2 Instituto de Ecología y Biodiversidad (IEB), Las Palmeras, 3425 Ñuñoa, Santiago Chile; 3 Laboratorio de Ecología Molecular, Departamento de Ciencias Ecológicas, Facultad de Ciencias, Universidad de Chile, Las Palmeras, 3425 Ñuñoa, Santiago Chile; 4 Department of Plant Biology and Ecology, Faculty of Science and Technology, University of The Basque Country (UPV/eHU), Leioa, Spain; 5 IKERBASQUE, Bilbao, Spain; 6 Departamento de Ciencias Biológicas y Químicas, Facultad de Recursos Naturales, Universidad Católica de Temuco, Temuco, Chile; 7 Núcleo de Investigación en Estudios Ambientales y Departamento de Ciencias Ambientales (facultad de Recursos naturales), Universidad católica de Temuco, Temuco, Chile; 8 Department of Ecology, IB, Universidade de Brasília, Brasília, Distrito Federal, Brazil; 9 Unidad de Cambio Climático y Medio Ambiente, Instituto de Estudios del Hábitat, Facultad de Arquitectura y Construcción, Universidad Autónoma de Chile, Temuco, Chile

Correction to: *Scientific Reports* 10.1038/s41598-020-58516-3 and 10.1038/s41598-019-44290-4, published online 30 January 2020 and 28 May 2019, respectively.

Due to a file provision error, the Publisher Correction for the original Article "Spatial distribution freshwater crustaceans in Antarctic and Subantarctic lakes" contained further errors in Table 1.

The endemic species (* or **), authority name and year of each species was omitted.

In addition, the authors detected taxonomic errors in the species *Acanthocyclops michaelseni* and *Tropocyclops prasinus*. *Acanthocyclops michaelseni* was synonymized to *Diacyclops michaelseni* and *Tropocyclops prasinus* was described by Pugh et al. (2002)^10^. *Tropocyclops prasinus* currently includes 12 subspecies, of which *Tropocyclops prasinus prasinus* is the subspecies described for the Falkland Islands.

As a result, under subheading “Cyclopoida”, species “*Acanthocyclops michaelseni*” should read: “*Diacyclops michaelseni* (Mrázek, 1901)**”

Under the same subheading, "*Tropocyclops prasinus*" should read: "*Tropocyclops prasinus prasinus* (Fischer, 1860).”

Table 1 legend,

“Presence/absence matrix of crustaceans in lakes of each study province (CA, Continental Antarctic; MA, Maritime Antarctic; SA, Subantarctic islands; SCT, Southern Cool Temperate), based on Pugh et al. 2002^10^ and Dartnall et al. 2017^6^.”

should read:

“Presence/absence matrix of crustacean taxa in lakes of each study region based on Pugh et al. 2002^10^ and Dartnall et al. 2017^6^. Provinces: CA, Continental Antarctic; MA, Maritime Antarctic; SA, Subantarctic islands; SCT, Southern Cool Temperate. Regions: En, Enderby; Wi, Wilkes; Sc, Scott; So, South Orkney Islands; Ss South Shetland Islands; Pa, Antarctic Peninsula; Sg, South Georgia; Pe, Prince Edward Island; Cr, Iles Crozet; Kr, Iles Kerguelen; Hd, Heard Island; Mc, Macquarie Island; Fa, Falkland/Malvinas Islands; Ca, Campbell Island; Ak, Auckland Island. Underlined species: Endemic from one biogeographic province; *Endemic from two or more biogeographic provinces; **Endemic from one region within a province.”

The correct Table 1 and accompanying legend appears below.

**Table 1:** Presence/absence matrix of crustacean taxa in lakes of each study region based on Pugh et al. 2002^10^ and Dartnall et al. 2017^6^. Provinces: CA, Continental Antarctic; MA, Maritime Antarctic; SA, Subantarctic islands; SCT, Southern Cool Temperate. Regions: En, Enderby; Wi, Wilkes; Sc, Scott; So, South Orkney Islands; Ss South Shetland Islands; Pa, Antarctic Peninsula; Sg, South Georgia; Pe, Prince Edward Island; Cr, Iles Crozet; Kr, Iles Kerguelen; Hd, Heard Island; Mc, Macquarie Island; Fa, Falkland/Malvinas Islands; Ca, Campbell Island; Ak, Auckland Island. Underlined species: Endemic from one biogeographic province; *Endemic from two or more biogeographic provinces; **Endemic from one region within a province.

**Order**/*Specie*		CA		MA			SA						SCT			Ref.
	En	Wi	Sc	So	Ss	Pa	Sg	Pe	Cr	Kr	Hd	Mc	Fa	Ca	Ak	
**Anostraca**																
*Branchinecta gaini* (Daday, 1910)				●	●	●	●						●			10, 6
**Cladocera**																
*Alona guttata* (Sars, 1862)**													●			10
*Alona quadrangularis* (O.F. Müller, 1776)**												●				10, 6
*Camptocercus aloniceps* (Ekman, 1900)*							●						●			10, 6
*Camptocercus rectirostris* (Schödler, 1862)							●									10
*Chydorus patagonicus* (Ekman, 1900)							●					●				10, 6
*Chydorus sphaericus* (O.F. Müller, 1776)*				●			●		●	●			●			10, 6
*Daphnia gelida* (Brady, 1918)**												●				6
*Daphnia pulex* (Leydig, 1860)													●			10
*Daphniopsis studeri* (Rühe, 1914)*	●							●	●	●	●					10, 6
*Ceriodaphnia silvestrii* (Daday, 1902)**													●			10
*Ilyocryptus brevidentatus* (Ekman, 1905)				●			●						●			10, 6
*Macrothrix boergeni* (Studer, 1878)**										●						6
*Macrothrix flagellata* (Smirnov & Timms, 1983)**												●				6
*Macrothrix laticornis* (Jurine, 1820)**													●			10
*Macrothrix ruehei* (Kotov, 2007)								●	●							6
*Macrothrix oviformis* (Ekman, 1900)*				●	●	●	●									6
*Macrothrix* sp. (Baird, 1843)*											●					6
*Ovalona weinecki* (Studer, 1878)*				●	●		●	●		●	●	●	●			6
*Pleuroxus macquariensis* (Frey, 1993)**												●				10, 6
*Pleuroxus wittsteini* (Studer, 1878)								●		●	●					10, 6
*Bosmina (Eubosmina) coregoni* (Baird, 1857)													●			10
**Podocopida**																
*Candona* sp. (Baird, 1845)							●									10, 6
*Chlamydotheca pestai* (Graf, 1931)**							●									10, 6
*Chlamydotheca symmetrica* (Vávra, 1898)**													●			10
*Cypretta* cf *seurati* (Gauthier, 1929)**												●				10, 6
*Eucypris corpulenta* (G. O. Sars, 1895)**										●						10, 6
*Eucypris fontana* (Graf, 1931)*				●			●									10, 6
*Eucypris virens* (Jurine, 1820)										●		●				10, 6
*Ilyodromus kerguelensis* (G.W. Müller, 1906)								●	●	●						10, 6
*Neocypridopsis frigogena* (Graf, 1931)*				●			●									10, 6
*Tanycypris* sp. (Triebel, 1959)							●									10, 6
*Candonopsis falklandica* (Vávra, 1898)**													●			10
*Newnhamia patagonica* (Vávra, 1898)**													●			10
**Calanoida**																
*Boeckella poppei* (Mrázek, 1901)*	●			●	●	●	●						●			10, 6
*Boeckella michaelseni* (Mrázek, 1901)*							●						●			10, 6
*Boeckella brevicaudata* (Brady, 1875)										●	●	●				10, 6
*Boeckella vallentini* (Scott T., 1914)*								●	●	●			●			10, 6
*Boeckella* sp. (Guerne & Richard, 1889)*			●													6
*Gladioferens antarcticus* (Bayly, 1994)**		●														10, 6
*Parabroteas sarsi* (Daday, 1901)*				●		●	●						●			10, 6
**Cyclopoida**																
*Acanthocyclops robustus* (Sars G.O., 1863)**										●						1,2
*Acanthocyclops vernalis* (Fischer, 1853)										●						1,2
*Diacyclops michaelseni* (Mrázek, 1901)**										●			●			1,2
*Diacyclops mirnyi* (Borutzky & Vinogradov, 1957)**	●	●											●			10, 6
*Diacyclops joycei* (Karanovic et al. 2014)**			●													6
*Diacyclops kaupi* (Karanovic et al. 2014)**		●														6
*Diacyclops walkeri* (Karanovic et al. 2014)**	●															6
*Mixocyclops crozetensis* (Kiefer, 1944)**									●							10, 6
*Paracyclops chiltoni* (Thomson G.M., 1883)									●	●						10, 6
*Tropocyclops prasinus prasinus* (Fischer, 1860)													●			10
**Harpacticoida**																
*Antarctobiotus koenigi* (Pesta, 1928)**							●									10, 6
*Antarctobiotus robustus* (Richters, 1907)									●	●						10, 6
*Epactophanes richardi* (Mrázek, 1893)								●	●	●	●					10, 6
*Marionobiotus jeanneli* (Chappuis, 1940)*								●		●		●				10, 6
*Marionobiotus* sp. (Chappuis, 1940)*												●				6
*Tigriopus angulatus* (Lang, 1933)*								●	●	●		●				10, 6
*Attheyella (D.) trigonura* (Eckman, 1905)													●			10
**Amphipoda**																
*Kergueleniola macra* (Ruffo, 1970)**										●						10, 6
*Pseudingolfiella possessionis* (Smet, 2015)**									●							6
*Chiltonia mihiwaka* (Chilton, 1898)														●	●	10
*Hyalella curvispina* (Shoemaker, 1942)													●			10
*Hyalella neonoma* (Stock & Platvoet, 1991)													●			10
*Falklandella obtusa* (Schellenberg, 1931)													●			10
*Praefalklandella cuspidatus* (Schellenberg, 1931)**													●			10
**Isopoda**																
*Iais* sp. (Bovallius, 1886)												●				6
Nº total species	4	3	1	9	4	4	17	9	11	19	6	14	25	1	1	
Nº species by zones	7			9			46						26			
Nº order	3			4			8						7			

